# A Treatise on Different Laborious Deliveries, and on Polypi of the Uterus

**Published:** 1782

**Authors:** 


II. *A Treatise on different laborious Deliveries, and on Polypi of the Uterus. By* M. Herbiniaux. (Continued from p. 170).

OF the firſt volume of this work we gave an account in our laſt number. In the ſecond volume the author treats of the uterine polypus. He begins with lamenting the inattention of practitioners to this diſeaſe, the very exiſtence of which, he obſerves, has been denied by ſome of the moſt eminent in their profeſſion. He oppoſes this erroneous opinion by remarking, that, as the uterus and vagina are covered with a membrane ſimilar to that which lines the noſtrils and other cavities where polypi are acknowledged to be frequently found, ſo it ſeems reaſonable to believe that the former are not exempt from their attack. He alſo quotes the authority of ſeveral authors, ancient as well as modern, who have deſcribed them under different names, and propoſed methods of eradicating them by the crotchet, fillet, &c. He thinks it is owing to a want of preciſion in the writers on this ſubject, and to the neglect of examining ſooner in painful and obſtinate diſeaſes of the uterus, that the diſorder in queſtion is ſo little known, known. He is of opinion that if an examination was conftantly and accurately to be made in fuch cafes, we fhould often find a polypus to be the unfufpected caufe of uterine hæmorrhage, or miftaken for cancers; and thus patients, deemed incurable and condemned for life to bear a naufeous difeafe, might, by an eafy operation, be reftored to health. He further remarks, that polypi are fometimes confounded with partial or total defcents of the uterus; and infifts, that in all the cafes in which the pendent uterus has been faid to be fafely removed by the knife or ligature, it has been fimply a polypus that has been thus extirpated.

From this general and indifcriminate cenfure of practitioners our author excepts the late M. Levret, from whofe large and ufeful collection of cafes and obfervations he recites feveral facts, which tend at once to fhew the frequency of the difeafe, and the almoft general ignorance of the profeffion on this fubject.

We are next prefented with a definition of the word Polypus, and a particular account of thofe treated of in this book. He defcribes them as a flefhy fubftance, varying in confiftence and bulk, and adhering to the uterus or vagina (from whence they draw their nourifhment) by a

ſingle pedicle or ſtalk more or leſs thick, but always conſiderably ſmaller than the body of the polypus. To this the author might have added from M. Levret, that they are covered with a membrane, which is a continuation of that which lines the cavity from whence they take their origin, and is plentifully ſupplied with arteries and veins, the latter being larger than the former, and in thoſe polypi, which ariſe from the fundus uteri, frequently varicous. Uterine polypi may be diſtinguiſhed, we are told, from a mole, by obſerving the breaſts of the patient, which are tumid as in time of pregnancy when there is a mole, but flaccid when there is only a polypus. The uterus, when diſtended with a mole, falls from ſide to ſide, according to the motion of the woman; but when there is a polypus, that viſcus is kept ſteady in its place; the abdomen alſo in the latter caſe is leſs enlarged. The os uteri, in caſe of a mole, never opens until the latter has acquired its deſtined bulk and is about to be excluded, which it at length is by the force of labour pains; but when a polypus is contained in the uterus, the os uteri continues dilated ſeveral months or years, without exciting any of thoſe convulſive throes, which in this caſe would ſooner terminate in the inverſion inverſion of the uterus than in the detachment of the offending body. M. Levret explains this by ſuppoſing that, as the increaſe of the polypus is gradual and almoſt inſenſible, it acts as a wedge on the os uteri, and thus by ſlow degrees procures a paſſage through that opening, without exciting thoſe contractions of the fundus uteri, which the ſudden and forcible action of a fœtus or mole upon that orifice would occaſion. It is this ſtricture of the os uteri round the body or pedicle of the polypus that occaſions the varicous appearances of the veins juſt now ſpoken of, and to the burſting of which M. Levret aſcribes the hæmorrhage that conſtantly attends thoſe which take their riſe from the fundus uteri; this ſtricture is ſometimes ſo conſiderable as to act as an artificial ligature, and occaſion the waſting and at length falling-off of the polypus; of which he quotes ſeveral examples from Mauriceau and others. Polypi, our author obſerves, may be diſtinguiſhed from a partial deſcent or inverſion of the uterus by introducing a finger into the vagina, becauſe if it is the uterus that deſcends it will be found to increaſe in bulk upwards, whereas the polypus tapers towards its pedicle or ſtalk. The inverted or deſcending uterus alſo, unleſs it has continued tinued till it is diſeaſed, may eaſily be returned and detained; while the polypus either cannot be put back, or at leaſt returns as ſoon as the reſtraining force is removed. In a total deſcent or inverſion of the uterus, the vagina being drawn with it, it is impoſſible to introduce a finger into the pelvis; but the polypus being attached to the internal ſurface of the uterus or vagina, allows the finger to paſs around its body, and frequently to trace it to its inſertion. A ſcirrhus, or occult cancer, may be diſtinguiſhed from a polypus by their ſenſibility, the latter, particularly at its baſis, being abſolutely inſenſible. Scirrhi alſo have no pedicles, but are broader where they are attached to the uterus, or vagina, than at their ſummit. From this account of the nature of uterine polypi, and of the marks by which they may be diſtinguiſhed from other diſeaſes of thoſe parts, the author proceeds to his method of extirpating them by ligatures, and to a deſcription of an inſtrument he has invented to carry the nooſe up to the neck of the polypus, when it is either ſeated ſo high, or the paſſage is ſo ſtrait as not to allow it to be performed by the fingers. The idea of this invention, which appears to be extremely ingenious, he acknowledges to have taken from

M. Lev-

M. Levret's; but contends, and feemingly with reafon, that it is much fuperior to the latter, particularly in the eafe and certainty with which it may be applied. But as this part of the work would not be intelligible without the engravings, which the author has annexed to this defcription, we muft refer our readers to the volume itfelf, and pafs on to fome reflections he has made on the operation. He obferves, that in many inftances this operation is not to be performed without confiderable pain, and even danger; and in one cafe, where the polypus was very large (nearly of the fize of a child's head at its full time, and the neck of it four inches in diameter) and its fubftance of a tendinous hardnefs, after feveral attempts to remove it by the ligature, he was obliged, on account of the extreme pain it occafioned, to untie the noofe. At length, however, he contrived to faw through the neck of the polypus by drawing a ftrong thread alternately backwards and forwards. How an operation of this kind could be fuccefsfully performed upon fo folid a body, placed fo far out of the reach of the operator's fingers, we confefs ourfelves unable to conceive. Several cafes are related in which the ligature was ufed, and which ferve to explain the nature of the the inſtrument, and the manner of applying the noofe. The work cloſes with an account of the approbation his methods have received from the Academy of Surgery at Paris and the Chirurgical Society at Amſterdam, as well as from ſeveral private and eminent practitioners. Upon the whole, although we are not diſpoſed to believe that the diſeaſe is ſo frequent as the author imagines, yet we cannot but acknowledge, that it may be ſometimes preſent when it is not ſuſpected, and muſt therefore confeſs ourſelves indebted to him for the cautions he has publiſhed on this ſubject.

His work is certainly highly deſerving the attention of practitioners, and to them we beg leave to recommend a trial of his inſtrument, as experience is the only teſt by which its merit can be decided.

